# Ethical dilemmas faced by healthcare teachers during the COVID-19 pandemic

**DOI:** 10.1177/09697330231215957

**Published:** 2023-11-24

**Authors:** Monika Koskinen, Yvonne Hilli, Tuulikki Keskitalo, Merle Talvik, Ann-Helen Sandvik, Kari Marie Thorkildsen, Maria Skyvell-Nilsson, Meeri Koivula, Jekaterina Šteinmiller

**Affiliations:** Åbo Akademi University; 1786 Nord University; 52914 Lapland University of Applied Sciences; 145045 Tallinn Health Care College; 1802 University of Borås; 1657 Western Norway University of Applied Sciences; University West; 7840 Tampere University; 145045 Tallinn Health Care College

**Keywords:** Caring science, COVID-19, ethical dilemma, healthcare teacher, hermeneutics, thematic analysis

## Abstract

**Background:**

Previous studies have shown that the rapid transition to emergency remote teaching due to the COVID-19 pandemic was challenging for healthcare teachers in many ways. This sudden change made them face ethical dilemmas that challenged their values and ethical competence.

**Research aim:**

This study aimed to explore and gain a deeper understanding of the ethical dilemmas healthcare teachers faced during the COVID-19 pandemic.

**Research design:**

This was an inductive qualitative study using a hermeneutic approach. Semi-structured interviews were conducted and analysed thematically.

**Participants and research context:**

Healthcare teachers (*n* = 20) from eight universities and universities of applied sciences in the Nordic and Baltic countries participated.

**Ethical considerations:**

This study was based on the research ethics of the Norwegian National Research Ethics Committee for Medicine and Health Sciences and approved by the Norwegian Agency for Shared Services in Education and Research.

**Findings:**

Healthcare teachers faced several ethical dilemmas due to restrictions during the COVID-19 pandemic. The analysis revealed three main themes: How should I deal with students’ ill-being, and what can I as a teacher do?; What can I demand from myself and my students, what is good teaching?; How do I manage the heavy workload and everyone’s needs, and who gets my time?

**Conclusions:**

This study highlights the importance of healthcare teachers’ continuous need for pedagogic and didactic education, especially considering new technology and ethical issues. During the pandemic, the ethical consequences of remote teaching became evident. Ethical values and ethical dilemmas should be addressed in healthcare education programmes at different levels, especially in teacher education programmes. In the coming years, remote teaching will grow. Therefore, we need more research on this issue from an ethical perspective on its possible consequences for students and healthcare teachers.

## Introduction

When the COVID-19 pandemic broke out, many healthcare teachers were forced to rapidly change their way of working, and there was insufficient time to reflect on how to maintain a high quality of education.^1^ This change entailed many challenges, including ethical dilemmas, and caused tremendous strain for teachers. Being a healthcare teacher means having a wide range of competencies that allow the adaptation of teaching to a variety of situations and individual students.^2,3^ The teacher’s ethical competence is particularly important when rapid changes lead to difficult ethical choices. Then, the teacher’s ethos becomes visible in ethical bearing and actions.^2–4^ In times of rapid change, it is essential to formulate and preserve ethical values.^2^

Ethical dilemmas arise when at least two ethical rules or obligations conflict and the outcome is undesirable, regardless of the chosen alternative.^5,6^ Therefore, ethical dilemmas force people to compromise between solutions in which all alternatives entail undesirable consequences.^7^ During the COVID-19 pandemic, there were challenges in implementing the teacher’s code of ethics in realising professional teaching.^8–10^

The authors of this article anticipate that healthcare educators’ mission to teach nursing using emergency remote teaching (ERT) and provide clinical practice for students raised unique ethical dilemmas due to the nature of nursing care and the fact that clinical practice was held in a field strongly affected by the COVID-19 pandemic. The complexity of conditions with a previously unknown, fatal disease, initially without clear prevention and treatment, was perceived as frightening, partly about how it affected the teaching of nursing care, partly in relation to students in clinical education, and partly their own fear of falling ill. The healthcare teachers had to prepare the students for new and unfamiliar educational and clinical situations and respond to the fear and uncertainty expressed by the students. At the same time, they were expected to do ‘business as usual’ as far as possible in teaching conditions that were unaccustomed to them. Furthermore, remote teaching continued after the pandemic, both in theoretical and clinical teaching. Therefore, it is essential to learn from healthcare teachers’ experiences concerning ethical dilemmas during the COVID-19 pandemic.

## Background

The outbreak of the COVID-19 pandemic created unprecedented economic, social, and political challenges around the world. Besides triggering a health crisis, it resulted in an education crisis, forcing the closure of educational institutions required by social distancing measures.^11–14^ This created a situation in which most educational programmes had to transfer instantly to ERT, which is different from planned online teaching, as it involves a temporary shift to an alternate teaching method due to crisis circumstances.^13^

During the lockdowns and quarantines, 87% of the world’s students were affected, and 1.52 billion learners were out of school and related educational institutions.^15^ Technology took a strong foothold in higher education classrooms, regardless of teachers’ and students’ preparedness for it. However, there were no other options then, and the changes had to be made quickly and in often chaotic circumstances. ERT was a fundamental solution to continue with academic curricula, with evident advantages and disadvantages.^16^

The theoretical perspective of this study is grounded in caring science, implying an ethos with caritas at its core (i.e. love, compassion, and respect for human dignity). Ethos implies an ‘inner ought’ in human beings, that is, the core values and moral attitudes that promote a good life. A person who is in touch with their core values is responsive to the voice of the heart and dares to be truly human and feels a sense of at-homeness, that is, belonging.^17,18^ Ethos indicates a person’s motive and driving force, and the basis for ethics and ethical actions is thus derived from our ethos.^18,19^ A relationship is determined by the nature of the involved persons’ ethical foundation, sense of responsibility, and willingness to invite others into a caring relationship. Caring means that the core of caring ethics is present and becomes visible and evident in the manner of conduct and the moral climate in the physical space in which students and healthcare teachers encounter and interact.^20^ Our ethos helps to create the culture and atmosphere of our relationships and interpersonal encounters,^19^ but if the culture changes, the value system will also change. The ethos of caring is the ethical framework within which the research question is defined and analysed.

## Research aim

This study aimed to explore and gain a deeper understanding of the ethical dilemmas healthcare teachers faced during the COVID-19 pandemic.

## Methodology and methods

This study has a qualitative design with an open-minded hermeneutic approach based on Gadamer’s hermeneutic philosophy.^21^ According to Gadamer,^21^ the process of understanding involves an attempt to capture the meaning of the whole through a continuous dialectical movement between the parts and the whole, the so-called hermeneutic spiral of understanding. The process continues until the preconceptions and understanding horizons of the interpreters’ merge with the text, and a shared meaning emerges. This study used thematic analysis to gain a deep understanding of participants’ experiences.

### Data collection

The data for this study were collected in 2021 through semi-structured interviews. Given the ongoing COVID-19 pandemic, the interviews were held via Microsoft Teams or ZOOM. In total, nine researchers conducted 20 interviews in Finland (*n* = 10), Estonia (*n* = 2), Sweden (*n* = 5), and Norway (*n* = 3) at universities and universities of applied sciences with senior and junior healthcare teachers. This study is part of a more extensive study exploring healthcare teachers’ experiences during the COVID-19 pandemic. The participating healthcare teachers, unknown to the researchers, were interviewed using an interview guide consisting of questions about teaching during the COVID-19 pandemic.

The primary interview question regarding ethical dilemmas was: *If you experienced ethical dilemmas as a healthcare teacher during COVID-19*, *please tell me what kind of dilemmas you encountered?* The concept of ethical dilemma was not defined in advance; the researchers relied on the participants’ pre-existing understanding. The participants were encouraged to develop their thoughts on the theme with follow-up questions, such as, ‘*Can you expand on your thoughts*?’ The interviews were conducted in the participant’s native language, recorded and transcribed verbatim in the language spoken during the interviews, namely, Swedish, Finnish, Estonian, or Norwegian.

### Data analysis and interpretation

Thematic analysis consistent with Braun and Clarke^22^ was used. Thematic analysis is about generating knowledge through the analysing process in a creative and interpretive way, moving back and forth between the different parts of the text and the whole to gain a deeper understanding.^22,23^ Six steps were involved in coding and thematisation: (1) familiarising oneself with the data, (2) generating initial codes, (3) searching for themes, (4) reviewing themes, (5) defining and naming themes, and (6) producing the report. To familiarise themselves with the data, the researchers read the interviews several times, making notes on their initial thoughts. The research question for the study guided the coding of the data, and both the conceptual and semantic coding of the material were conducted. All codes related to ethical dilemmas were identified. The codes were interpreted and arranged into potential sub-themes according to how they related to each other and how similarities and variations emerged in the data material. Author’s xx and xx were responsible for the data analysis and mastered the languages except Estonian. When analysing the Estonian interviews, the data were translated into English by the Estonian researchers.

All researchers, that is, all authors, met several times to reflect on the sub-themes and themes until a consensus was reached. The process of analysis was challenging because the data was in different languages. Therefore, it was important for all researchers to meet regularly to avoid misinterpretations.

Further reading of the coded extracts was conducted to verify that the sub-themes formed were relevant; the sub-themes were arranged and clarified to highlight the core of each theme. In the next step, the sub-themes were reviewed in relation to the whole. Certain sub-themes were merged, and three main themes were formed. In the next phase, the themes were defined and named based on their essence and content. In the last phase, a report was written to describe the findings. The results were based on 51 codes (see Appendix) analysed into nine sub-themes and three themes (see Table 1).

**Table 1.** List of sub-themes and main themes.

Sub-themes	Main themes
Seeing students suffering but not knowing how to help.	How should I deal with students’ ill-being, and what can I as a teacher do?
To ask how students are doing or not to ask.	
Responsibility in relation to student’s ill-being.	
Rearranging teaching to ERT but no time to learn remote teaching didactics.	What can I demand from myself and my students, what is good teaching?
Maintaining high standards of performance or lowering the bar due to the difficult situation.	
Feeling of inadequacy in relation to the new, imposed, teaching methodology.	
Struggling to find balance between heavy workload and own well-being.	How do I manage the heavy workload and everyone’s needs, and who gets my time?
Problem of knowing how flexible you need to be in this situation.	
Private space occupied by work when working from home.	

## Ethical considerations

This study was based on the research ethical guidelines of the Norwegian National Research Ethics Committee for Medicine and Health Sciences (NEM 2019).^24^ The study was approved by the Norwegian Agency for Shared Services in Education and Research (Sikt: ref.nr. 501,217). Approval was also obtained from the faculty management at the universities participating in the study. All participants were contacted by e-mail and given written and oral information before the interview, including information on the study design, as well as assurance of anonymity, confidentiality and the ability to withdraw their participation at any time. In addition, the participants were informed that the interviews would be recorded, and they were briefed on the themes and estimated duration of the interviews. They confirmed their participation by signing consent forms before the interviews. All data were immediately anonymised so that no personal information could be connected to the data. Anonymity was ensured by giving all participants a number code (P1–P20).

## Findings

The findings of this study show that participants faced several ethical dilemmas due to restrictions during the COVID-19 pandemic. The analysis revealed three main themes: How should I deal with students’ ill-being, and what can I as a teacher do?; What can I demand from myself and my students, what is good teaching?; How do I manage the heavy workload and everyone’s needs, and who gets my time? The themes are questions arising from the ethical dilemmas the participants faced when switching to digital teaching due to the pandemic. The participants felt that they were presented with somewhat impossible choices in the shift from ordinary to remote teaching.

### How should I deal with students’ ill-being, and what can I as a teacher do?

The first theme expresses ethical dilemmas that concern the difficulty in addressing the student’s ill-being, their potential need for help, and whether it was the teacher’s responsibility to intervene. Previously, when meeting students in the classroom, an assessment of students’ well-being could be made based on their body language, eye contact, and responses, making it easier to determine the need for intervention. Digitalisation essentially removed this possibility and made it more challenging to make the right decisions. Asking directly about a student’s well-being was sometimes perceived as violating the student’s privacy. However, being worried and not asking was contrary to the participant’s inner values. ‘You feel that you need to help those who are having a hard time in this situation, but you don’t know how’ (P16).

When teaching about nursing care, you deal with difficult topics. Being unable to be with the students when these topics were discussed and without sufficient time to prepare for distance learning made it more challenging to ensure that the students were given enough time and opportunity to learn. The participants felt that students were left alone after teaching difficult subjects, which could trigger questions and raise anxiety. Teaching these subjects raised questions about whether it was ethically correct not to offer time for necessary reflections. Were they possibly causing unnecessary suffering for students?

The physical meeting is needed to get a feeling of the other person’s reaction to what you say. If it happens [when using ERT] that the other gets sad and starts crying, then it is much more difficult for me to somehow be present and show my compassion. (P2)

The participants encountered loneliness and various forms of anxiety among the students. Some students attended class from their beds, perhaps not even dressed. An ethical dilemma concerned whether demanding students should have the camera on or allowing them to choose when to use the camera. For teaching and interaction, it would be good for students to have the camera on, but if they did not feel well, the participants did not want to force them to put the camera on. As a result, the participants wondered whether it was their responsibility to consider the student’s well-being or whether they should demand a camera regardless of how the student was performing. Not knowing how to act in these situations created an internal dilemma.

I don’t know if this is directly an ethical dilemma, but the fact that I don’t see my students, I don’t see how they feel. I can’t give everything that I would like to give and would normally be able to do to help. (P18)

According to the participants, the students struggled considerably during the pandemic, and some had difficulty coping with remote studies and the pandemic in general. The particular situation of the pandemic raised questions about how they, as teachers, should respond to students’ ill-being, in teaching situations but also outside the classroom. Is the teacher responsible for intervening when students were unwell?

Sometimes, I felt that there were ethical dilemmas related to the students who I noticed were feeling very bad. Getting hold of them and contacting them during the coronavirus pandemic required a lot of work, while at the same time, I thought about how much I can do and what is my role as a teacher. (P17)

In addition to concerns about students’ ill-being, participants encountered ethical dilemmas related to student clinical placements. There was concern about the safety of students working with COVID-19-infected patients. Had students been adequately taught about infectious diseases such as COVID-19? A concern about their spreading the virus when moving from one place to another. An ethical dilemma was the concern for the safety and health of students in clinical practice. Teachers did not have the mandate to decide because decisions were made at a higher level.

### What can I demand from myself and my students, what is good teaching?

The second theme describes ethical dilemmas that arose when participants who considered themselves to be good teachers could not facilitate teaching based on their values. In a short time, essentially overnight, teaching had to be changed from the familiar classroom to remote teaching, not familiar to many. This constraint was very challenging, as many participants did not feel equipped with enough didactic knowledge and did not have time to prepare. It also presented an ethical dilemma concerning teachers’ professional identity. Participants tried their best to adapt their classroom to remote teaching and find different strategies to solve the dilemmas.

One strategy has been to set the bar lower than I might otherwise have had it, but at the same time, it’s sometimes even harder. But I just have to realise that I cannot do everything at once, and I must choose what I do. (P17)

The participants knew there was no other option than ERT given the COVID-19 pandemic. However, this did not change the fact that teaching and student learning were affected by the transition. A significant challenge was the unique nature of nursing education, in which face-to-face teaching plays a vital role in promoting the development of a caring attitude in students. Reflecting on difficult topics, seeing how students reacted to what was taught, and assessing how students’ learning progressed became more difficult. A major source of stress and ethical dilemmas was not being able to guarantee effective teaching and sometimes having to give up even what was considered fundamental in the teaching of nursing care.

In the training to become a nurse, it is so incredibly important to be in an encounter with another human being. It is down to an existential level where it is about something other than a relationship. To meet can mean so much, and words can be misunderstood. It is about listening and giving and receiving and enduring silence together. To be in silence is much more difficult to train when you have seminars in Zoom. (P5)

Ethical dilemmas also occurred with didactical issues and the assessment of students’ learning. As one participant put it: ‘Especially the professional interaction that should develop while studying here is impossible to teach online’ (P12). Some participants felt they were not qualified to teach clinical skills, critical thinking, and decision-making at a distance, areas of healthcare education considered particularly important.

For me, teaching is an interaction that goes both ways. Now, I do my monologue, and although I really like to talk about the subject, I don’t think it gives the best learning opportunity for the students. I think interaction is crucial in teaching and that we create knowledge together. (P1)

The participants expressed ethical dilemmas related to maintaining high standards of performance or lowering the bar because of difficult situations. They pondered how much workload they could impose on students to meet the learning objectives while also considering the students’ difficult situations coping with the pandemic. ‘You can’t demand too much, but you have to demand enough’ (P15).

There were concerns about their ability to teach remotely and how it would affect students’ motivation to achieve learning outcomes. ‘There were some thoughts about whether they get quality teaching through this remote teaching and how we can keep up that motivation in their studies’ (P10). This sometimes created a sense of inadequacy; no matter how hard the teacher tried, they felt it was never enough for student learning. The ethical dilemma was about whether ‘good enough’ was enough or whether they should try even harder. Responsibility weighed heavily, but reality made it difficult to live up to their own and others’ expectations.

It has required an incredible amount of time, and I’m not happy with everything, definitely not. I would have liked to be able to spend more time on the planning and creation of the lessons than I have had. (P17)

### How do I manage the heavy workload and everyone’s needs, and who gets my time?

The third theme revealed that participants encountered ethical dilemmas in choosing how they would deal with their lack of time and the need for perseverance. They felt responsible for their families’ well-being but also for their work, colleagues, and students. A general lack of time contributed to their difficulties in prioritising significant tasks. ‘There is not enough time for the demands that are put upon us all the time’ (P12).

In the initial phase of the pandemic, participants felt that students often needed more help with their studies and technical issues. Moreover, all the extra precautions related to the pandemic were energy consuming. The participants had families with their own needs and often struggled to meet the family’s needs, while many things demanded their attention.

Colleagues often had divergent opinions regarding how flexible they should be, which led to feelings of unequal treatment of students. This created a dilemma for participants who wanted to show collegiality and not demand more or less from students than the others did. At the same time, they felt that their colleagues’ choices did not match what they felt was right in some situations.

The situations when you must be flexible and tailor those individual things and solutions, and there is no consensus between the teachers on how much we should flex. Some of us are ready to flex more, some much, much less, so the dilemma then comes in what the interface is and how much to flex. (P11)

Another ethical dilemma was how to get rest and detach oneself from work. The participants experienced that work and private life melded into each other when teaching moved into their homes. The whole situation with the pandemic, worries of different kinds, and an unfamiliar way of teaching and handling new situations created considerable ethical stress. The participants expressed feelings of inadequacy and fatigue. ‘*I just tried to cope*’ (P18) was one participant’s recollection of teaching during the pandemic’s first phase. They felt the need to set boundaries, but feared the students would not learn what was expected. On the other hand, working without boundaries was too demanding.

I still feel that it is my ethical duty to be there for those students. But I feel that there is an ethical dilemma here, that the teacher must be there for the students and must help them and listen to them and be empathetic. But this is not considered in our working hours by the management. (P16)

The participants’ families and their social lives were affected by the pandemic. Many had children, even toddlers, at home when working, which directly affected their ability to work. This affected their concentration, and in some cases, confidentiality was threatened. The ethical dilemma was the need to juggle many different tasks to get the job done while maintaining focus.

The participants felt great loyalty to their employers. Still, the situation and its effect on their private lives sometimes became burdensome, and they did not know to whom they should be most loyal: themselves and their families, their students, their colleagues, or their employers. ‘And yes, in my role I try to do my best, but I don’t feel like I’m good enough. There simply isn’t room in my schedule for what they might want’ (P4).

## Discussion

This study aimed to better understand the ethical dilemmas healthcare teachers faced during the COVID-19 pandemic. The study demonstrates that healthcare teachers faced different ethical dilemmas when familiar classroom teaching suddenly had to be changed to digital teaching due to the COVID-19 pandemic. The teachers asked themselves what they would do when they could not teach according to their values. Was it possible to teach digitally in a way that still yielded an acceptable, if not the best, result? According to Hilli and Eriksson,^20^ people in touch with their fundamental values feel metaphorically at home. It means feeling safe and confident, being able to act according to one’s own conscience and what is the right thing to do. During the pandemic, both teachers and students were expected to continue working and studying despite the circumstances, which previous studies^25–27^ show created a sense of uncertainty and insecurity, ill-being, a kind of homelessness.^20^ The findings in this study show that healthcare teachers often felt lost in how to ethically address the situations that arose. They also noted that closed campuses and not having classmates to socialise with gave students a sense of not belonging anywhere, which can be understood as a form of homelessness contributing to students’ ill-being.

When digital teaching moved to the teachers’ homes, the situation caused a disruption that affected their professional and personal identity, causing feelings of loss of control, an inability to meet their responsibilities as teachers, and a concern that the students were suffering. Ethical values are a cornerstone for teachers to create a culture and atmosphere for relationships and interpersonal encounters^19^; however, if the hierarchy of values changes, the culture will also change. This study shows that healthcare teachers, despite chaotic conditions, could navigate through the demands and keep their focus on the primary goal. The participants strived to create an ethical atmosphere in the learning environment where students and healthcare teachers met and interacted.^20^

The dilemmas this study identified are understood in relation to the uniqueness of healthcare teachers’ values and ethical competence.^4^ Their values, or ethos, became evident in their manner of conduct. Being unable to act according to one’s values gives rise to ethical dilemmas and moral stress, as described by Colnerud.^28^ The healthcare teachers’ narratives testify to their experience of moral stress. The dilemma between their values and the reality faced was difficult to resolve.^18,20^

Remote teaching is challenging for communication and approachability. According to Vollmann et al.,^29^ students experienced a decline in study-related well-being, study effort, and education satisfaction when the lockdown allowed only remote teaching. Caring for students’ well-being was a significant concern for healthcare teachers. Remote teaching was a somewhat new method, and teachers were concerned about whether their teaching was good enough and how much they could demand of the students to accomplish the learning objectives.

Due to the rapid spread of the pandemic, healthcare teachers were constantly presented with new information and guidelines about teaching arrangements, creating different kinds of dilemmas. Sarkar et al.^30^ found that a change in teachers’ pedagogic philosophy was required when teaching remotely. The teachers in the Sarkar et al.^30^ study felt less ‘organic and responsive’ when forced to teach remotely and lacked knowledge in distance learning. This finding is consistent with our study, in which healthcare teachers expressed not being sufficiently skilled when moving to remote teaching without adequate education in the didactics of distance teaching and learning.

The healthcare teachers in this study felt they were largely left on their own to find new ways to teach clinical skills, critical thinking, and decision-making remotely. Ethical dilemmas sometimes arose when exploring alternative assessment methods to maintain academic rigour while being sensitive to the ill-being of their students. There were many technical challenges, but very little time to re-evaluate the methodological concepts of teaching or to thoughtfully reorganise work. These constraints were also noted by Rizun and Strzelecki,^31^ who highlighted the importance of not interrupting the teaching and learning process. Unclear instructions by administrations, blindness to the student situation and capacity, and complex home environments deepened the dilemmas for teachers, which Mäkelä et al.^32^ also contend. A recent study by Heikkilä et al.^33^ found that ethical dilemmas have a significant impact on teachers’ well-being. There are similar ethical dilemmas in our findings, in which teachers struggled with feelings of inadequacy about work and their personal lives.

## Strengths and limitations

The strength of this study is that it was conducted in several countries, providing a meaningful picture and a deeper understanding of how healthcare teachers experienced ethical dilemmas during the pandemic. An interview guide was used to ensure the uniformity of the interviews. A limitation might be that the concept of ethical dilemmas was not defined in advance, resulting in different meanings for healthcare teachers in different cultures. Only female teachers participated, which may have influenced the results and made them more ambiguous in relation to ethical dilemmas. Therefore, the application of the study’s results should be made with some caution.

## Conclusions

This study highlights the importance of healthcare teachers’ continuous need for pedagogic and didactic education, especially considering new technology and related ethical issues. During the pandemic, the ethical consequences of remote teaching became evident. The employer responsible for the employee’s health and well-being should be aware of the moral stress ethical dilemmas may cause teachers. Therefore, it is essential that the employer facilitates further education on ethical issues and regular meetings to address possible ethical dilemmas and promote the well-being of teachers. Ethical values and ethical dilemmas should be addressed in healthcare education programmes at different levels, especially in teacher education programmes. In the coming years, remote teaching will continue to grow. Therefore, more research about this issue from an ethical perspective and its possible consequences for students and healthcare teachers is imperative.

## Supplemental Material


Supplemental Material - Ethical dilemmas faced by healthcare teachers during the COVID-19 pandemic
Supplemental Material for Ethical dilemmas faced by healthcare teachers during the COVID-19 pandemic by Monika Koskinen, Yvonne Hilli, Tuulikki Keskitalo, Merle Talvik, Ann-Helen Sandvik, Kari Marie Thorkildsen, Maria Skyvell-Nilsson, Meeri Koivula and Jekaterina Šteinmiller in Journal of Nursing Ethics.
