# Supplementary material for: Ethical dilemmas faced by healthcare teachers during the COVID-19 pandemic
Source: Nurs Ethics. 2023 Nov 24;31(5):907–18. doi: 10.1177/09697330231215957 (PMC11370175; doi:10.1177/09697330231215957)
Supplement: Supplemental Material - Ethical dilemmas faced by healthcare teachers during the COVID-19 pandemic [file sj-pdf-1-nej-10.1177_09697330231215957.pdf]

Appendix: List of codes, subthemes, and main themes

Codes	Subthemes	Main themes
- Difficult to identify students with problems due to ERT.<br>- Seeing students suffer from loneliness and not being able to help or knowing if to help.<br>- Teacher more forgiving because students are not feeling well but they know they should demand more.<br>- Need to be more sensitive for students' wellbeing even if even if it negatively affects teaching.<br>- Leaving students alone with difficult topics or not addressing the subjects.<br>- Not being able to help students as much as they need help does not feel good.<br>- Does not have knowledge how to support students during these circumstances and does not have time to learn.	Seeing students suffering but not knowing how to help	How should I deal with students' ill-being, and what can I as a teacher do?
- Hard to know if students are struggling without being able to ask in a discrete way.<br>- The distance makes it difficult to confirm the students even if you know they need it.<br>- Students are behind the black screen, ask them to turn on camera or not?	To ask how students are doing or not to ask.	
- COVID-19 pandemic overall a difficult time for students and it is hard to know what teacher's responsibility is in this case.<br>- Students don't get help anywhere else, teachers feel they must help even if they do not have time or possibility.<br>- Not being able to arrange meeting places for students.<br>- The students in more need of study help but dilemma for teacher finding time to help.<br>- Students taking care of Covid-19 patients in clinical practice or not letting them do clinical training resulting in prolonged education	Responsibility in relation to student's ill-being.	
- Demands on teaching are high, but resources are lacking.<br>- Core values in teaching at stake when compromises are made to make it work.<br>- Not meeting and interacting effects learning outcome.<br>- Reflection in teaching suffers	Rearranging teaching to ERT but no time to learn remote teaching didactics.	What can I demand from myself and my students, what is good teaching?

when technical issues make it more difficult, but what to do when ERT is necessary.<br>- Higher threshold for asking questions when not meeting face to face.<br>- Difficulties in recognizing students' reactions to teaching.<br>- Technical issues and problems take time and focus from content.<br>- Need to use technology for data transfer and teaching without being able to guarantee data security.<br>- Struggling with teaching communication and empathy online.	.	
- Poorer overview of students' knowledge.<br>- Harder to give nonverbal feedback.<br>- The need for control vs trust that the students do what they are supposed to.<br>- Teachers holds the bar but gets stressed or lowers the bar and feels bad.<br>- How much guidance is enough.<br>- Independent learning without teacher guidance requires trust and accurate assessment methods.<br>- Respond to students lack of motivation due to ERT or not.<br>- Dilemma to know how much load to put on student in a difficult time.<br>- How much control should the teacher have and how much responsibility should students take?<br>- Being flexible and adjust or stick to the requirements.	Maintaining high standards of performance or lowering the bar due to the difficult situation.	
- Feeling of inadequacy when not having the skills to teach remotely.<br>- Struggling with keeping quality or making it work.<br>- Feeling of inequality when some students can handle technology better than others, hard to know how to make it more equal.	Feeling of inadequacy in relation to the new, imposed, teaching methodology.	
- A lot of extra work due to ERT and need to take care of oneself and the family.<br>- Need to be present in multiple places at the same time, at work, home and in other places.<br>- Helping students after work hours or taking time to relax.	Struggling to find balance between heavy workload and own well-being.	How do I manage the heavy workload and everyone's needs, and who gets my time?

- Huge need for working overtime but that take away time for recovery.<br>- To know that students need help, but you also must rest.		
- Just trying to cope even if you have reached the limit of what you can do.<br>- Working without breaks to get the work done or taking breaks and not doing a god job.<br>- Trying to take care of own and students' well-being, hard to prioritize right.<br>- Feeling responsibility for new teachers and new students but do not have time to introduce them in a good way.<br>- A lot of questions you cannot answer.	Problem of knowing how flexible you need to be in this situation.	
- Hard to detach oneself from work, not easy to end work when working from home.<br>- Family and private time suffering due to stress and heavy workload.<br>- Difficult to set boundaries when there is need for much help in many places.<br>- Feeling that work life takes away from time with family.	Private space occupied by work when working from home.	
